# Sulphonylurea Usage in Melioidosis Is Associated with Severe Disease and Suppressed Immune Response

**DOI:** 10.1371/journal.pntd.0002795

**Published:** 2014-04-24

**Authors:** Xiang Liu, Geraldine Foo, Wan Peng Lim, Sharada Ravikumar, Siew Hoon Sim, Mar Soe Win, Jessamine Geraldine Goh, Joan Hui Juan Lim, Ying Hui Ng, Dale Fisher, Chin Meng Khoo, Gladys Tan, Louis Yi Ann Chai

**Affiliations:** 1 Division of Infectious Diseases, University Medicine Cluster, National University Health System, Singapore; 2 Department of Pharmacy, National University Hospital, Singapore; 3 Department of Pharmacy, Tan Tock Seng Hospital, Singapore; 4 Defence Medical and Environmental Research Institute, DSO National Laboratories, Singapore; 5 Faculty of Medicine, National University of Singapore, Singapore; 6 Division of Endocrinology, University Medicine Cluster, National University Health System, Singapore; University of California San Diego School of Medicine, United States of America

## Abstract

**Background:**

Melioidosis is a problem in the developing tropical regions of Southeast Asia and Northern Australia where the the Gram negative saprophytic bacillus *Burkholderia pseudomallei* is endemic with the risk of fulminant septicaemia. While diabetes mellitus is a well-established risk factor for melioidiosis, little is known if specific hypoglycemic agents may differentially influence the susceptibility and clinical course of infection with *B. pseudomallei* (Bp).

**Methodology/Principal Findings:**

In this cohort study, patients with pre-existing diabetes and melioidosis were retrospectively studied. Outcome measures: mortality, length of stay and development of complications (namely hypotension, intubation, renal failure and septicaemia) were studied in relation to prior diabetic treatment regimen. Peripheral blood mononuclear cells (PBMC) from diabetic patients and healthy PBMC primed with metformin, glyburide and insulin were stimulated with purified Bp antigens *in vitro*. Immune response and specific immune pathway mediators were studied to relate to the clinical findings mechanistically. Of 74 subjects, 44 (57.9%) had sulphonylurea-containing diabetic regimens. Patient receiving sulphonylureas had more severe septic complications (47.7% versus 16.7% p = 0.006), in particular, hypotension requiring intropes (p = 0.005). There was also a trend towards increased mortality in sulphonylurea-users (15.9% versus 3.3% p = 0.08). *In-vitro*, glyburide suppressed inflammatory cytokine production in a dose-dependent manner. An effect of the drug was the induction of IL-1R-associated kinase-M at the level of mRNA transcription.

**Conclusion/Significance:**

Sulphonylurea treatment results in suppression of host inflammatory response and may put patients at higher risk for adverse outcomes in melioidosis.

## Author Summary

Melioidosis is a problem in the developing tropical regions of Southeast Asia and Northern Australia where the Gram negative bacillus *Burkholderia pseudomallei* can cause life-threatening infection. Diabetes mellitus is a recognised risk factor for melioidiosis; however little is known if commonly used anti-diabetic drugs may affect the clinical course of the disease. In this study, we found that patients who were receiving sulphonylureas for diabetic treatment had more severe septic complications requiring intensive care as well as increased risk of deaths. This may be attributable to the capacity of sulphonylureas in modulating the host immune response. We highlight caution in the prescription of this class of drug, which is popular due to its low cost and easy availability, especially in melioidosis-endemic tropical regions.

## Introduction

Melioidosis is an environmental saprophyte endemic in tropical regions. The infection is caused by the Gram negative saprophytic bacillus *Burkholderia pseudomallei* found commonly in environmental soil and surface water [1]. Clinical presentations of melioidosis include bacteremia, abscesses in any organ systems, pneumonia or soft tissue infection. The clinical course can be mild and chronic through to fulminant leading to septic shock and death [2].

Diabetes mellitus is a well-established risk factor for susceptibility to melioidosis [3], [4]. Approximately 50% of patients presenting with melioidosis are diabetics, the majority of whom are already receiving anti-diabetic therapy. However little is known if specific hypoglycemic agents, by themselves, may differentially influence the susceptibility and clinical course of infection with *B. pseudomallei*. This is important in light of recent knowledge that sulphonylureas (glyburide) and the biguanides (metformin) may possess modulating capabilities on host immune response [5], [6]. It is known that the activation of host immune defence and the elaboration of inflammatory cytokines against the pathogen is requisite [7], [8] but opinion on the optimal cytokine milieu for facilitating an effective host response during melioidosis remains unclear [9], [10].

Hence we aim to study whether commonly prescribed anti-diabetic medications, in particular the sulphonylureas by virtue of their ascribed immunomodulating potential, may influence outcomes in melioidosis. Consequently, we also will attempt to underline the mechanisms by which the specified drug may exert their effects on host immune response against *B. pseudomallei*.

## Materials and Methods

### Cohort study

The study was conducted at two of Singapore's largest hospitals, the National University Hospital and the Tan Tock Seng Hospital, each with more than1000-beds capacity. We identified patients diagnosed with melioidosis between January 2001 to December 2010 from the integrated hospital electronic medical record systems. A total of 116 such patients were identified, of which 15 subjects (12.9%) did not have diabetes by history and laboratory investigation, while 27 subjects (23.3%) were newly diagnosed diabetics upon presentation with melioidosis. The remaining 74 patients (63.8%) had pre-existing type II diabetes and receiving medication prior to presenting with melioidosis and this was the study cohort being analysed.

All patients received the recommended melioidosis treatment regimen consisting of an initial intensive therapy consisting of either ceftazidime, meropenem or imipenem intravenously for at least 2 weeks, followed by a prolonged oral eradication phase consisting of a combination of 2 oral agents from the 3 antibiotics: trimethoprim-sulfamethoxazole (TMP-SMX), amoxicillin-clavulanate, doxycycline for at least 10 weeks [11]. Trimethoprim-sulfamethoxazole was the backbone of the 2-drug eradication regimen unless there were drug tolerance issues with individual patients. The patients were followed up for at least 12 weeks. Records of pre-existing diabetic medications were obtained from clinical notes and/or verified from computerised outpatient pharmacy archives. Most of the glycosylated haemoglobin (HbA1c) levels were measured upon admission to hospital with melioidosis. When this was not available, the last known HbA1c level (up to 3 months prior presentation) was traced through electronic outpatient records. The primary outcome measure was mortality. The secondary outcome measures included length of hospital stay and measures of disease severity, namely: (i) hypotension requiring the infusion of inotropes (ii) respiratory distress requiring mechanical ventilation (iii) renal impairment with need for renal replacement therapy (iv) septicaemia as per defined by the Surviving Sepsis Campaign Guidelines [12].

### Reagents

The Toll like receptor (TLR) 4 ligand lipopolysaccharide (LPS-EK Ultrapure, *Escherichia coli* serotype K-12) was purchased from Invivogen (San Diego, CA). Metformin (a biguanide), glyburide (a sulphonylurea) and human recombinant insulin were purchased from Sigma-Aldrich (Singapore) and re-suspended in DMSO. The antibodies ERK 1&2 (H-72) and p-ERK (E-4) were from Santa Cruz Biotechnology, Inc (Dallas, TX).

### Preparation of purified Bp antigen

Overnight culture of *B. pseudomallei*, Bp22, was inoculated into LB broth (Difco Laboratories, Detroit, Michigan) and incubated for 4 h at 37°C until an OD600 reading of 0.8–1.0. The bacteria was pelleted at 4000 g for 10 min and washed twice with 1X phosphate buffer saline (PBS), prior to reconstitution in 1X PBS containing 0.2 µg/ml leupeptin (Sigma-Aldrich, St Louis, MO), 0.2 µg/ml pepstatin A (Sigma-Aldrich, St Louis, MO) and 2.5 Kunitz units of DNase I (Sigma-Aldrich, St Louis, MO). To extract bacterial proteins, the suspension was loaded into lysing Matrix B tubes (MP Biochemicals, Solon, OH), and three rounds of homogenization was performed using Fastprep instrument (MP Biochemicals, Solon, OH) at a speed of 6 m/s for 30 s with a pause of 10–15 min in between for cooling. The bacterial lysate was centrifuged at 13000 g for 2 min, and passed through 0.22 µm filter unit (Merck Millipore, Billerica, MA) to remove any live bacteria. The supernatant was further purified and concentrated using Amicon Ultra-15 centrifugal units with 10 kDa nominal molecular weight limit (NMWL) membrane (Merck Millipore, Billerica, MA). The final purified Bp antigen were recovered in 1XPBS, quantified by BCA protein assay kit (Pierce Biotechnology, Rockford, IL) and used for further stimulation experiments.

### *In-vitro* stimulation assays

Separation and stimulation of peripheral blood mononuclear cells (PBMC) from healthy volunteers and diabetic patients was performed as previously described [13]. Blood was drawn into sodium heparin tubes (BD Vacutainer Franklin Lakes, NJ) after informed consent. PBMC were isolated by density centrifugation on Ficoll-Hypaque (Pharmacia Biotech, Uppsala, Sweden). Cells were washed, counted and adjusted to 5×10^6^ cells/mL in culture medium (RPMI 1640 DM supplemented with gentamicin, L-glutamine, and sodium pyruvate). Stimulation assays were performed in 96-well round-bottom plates using 100 µl of PBMC with the various stimuli and drugs to a total volume of 200 µl per well. PBMC were pre-incubated with metformin, glyburide, insulin or control media for 1 hour after which purified Bp antigen was added. After 24 h or 48 h of incubation in humidified atmosphere (5% CO2) at 37°C, the supernatants were collected and stored at −20°C until further assay.

### Cytokine assay

Interleukin(IL)-6, IL-10, IL-1β, tumour necrosis factor-alpha (TNF-α) and interferon- gamma (IFN-γ) were measured by commercial ELISA kit according to the instructions of the manufacturer (eBioscience, San Diego, CA). Detection limits were 20 pg/ml (IL-1b, TNF-α and IFN-γ) and 10 pg/ml (for IL-6 and IL-10) respectively.

### Western blot

PBMCs from healthy volunteers were treated with glyburide, metformin, insulin and the carrier medium (DMSO) for 1 h, followed by stimulation with purified Bp antigen for 30 mins. Whole-cell lysates were prepared in RIPA buffer and protein content was determined by the Bradford assay (Bio-Rad). 25 µg of lysates were electrophoresed on 10% polyacrylamide gels and transferred to polyvinylidene difluoride membranes. After blocking in 5% non-fat dry milk, the membranes were incubated with p-ERK (sc-7383), p-JNK (#4668) or p-p38 (#9215) primary antibodies. Horseradish peroxidase-conjugated secondary antibody was used and immunoreactive proteins were visualized with chemiluminescence detection substrate (Pierce). The membranes were stripped and re-probed for total ERK (sc-292838), JNK (#9252) and p38 (#9215). Western blot was also performed for MyD88 (sc-11356). Beta-actin (sc-47778) was used as the loading control. Antibodies from Santa Cruz Biotechnology or Cell Signaling were used.

### Flow cytometry

PBMC were incubated with glyburide, metformin, insulin and the carrier medium (DMSO) for 2 h prior to stimulation with purified Bp antigen for 30 minutes. Following stimulation, cells were washed twice with 1XPBS supplemented with 0.2% (w/v) BSA and stained with anti-CD14-V450 antibody (BD Biosciences, San Jose, CA). Cells were then fixed with BD Cytofix (BD Biosciences, San Jose, CA) and permeabilized using Perm III buffer (BD Biosciences, San Jose, CA) for 30 minutes. The permeabilized cells were stained with mouse anti- p-ERK1/2 antibody (BD Biosciences, San Jose, CA), mouse anti- phospho-JNK antibody (Cell signaling Technology, Danvers, MA) and goat anti-IRAK-M antibody (Santa Cruz Biotechnology, Dallas, TX). For cells stained with unlabelled goat anti-IRAK-M antibody, secondary labeling with donkey anti-goat-FITC antibody (Santa Cruz Biotechnology, Dallas, TX) was carried out. Stained cells were then analyzed on FACSCanto flow cytometer (BD Biosciences, San Jose, CA) and data analysis was performed using Flow Jo software (version 7.6.5) (Tree Star, Ashland, OR).

### Quantitative PCR

The expressions of interleukin-1 receptor-associated kinase-1(IRAK-1) and interleukin-1 receptor-associated kinase-M (IRAK-M) in PBMC primed with glyburide, metformin, insulin and control medium were determined. RNA was extracted from 10^7^ PBMC by using 1 ml TRIzol reagent (Sigma, St. Louis, MO). Subsequently, 200 µl chloroform and 500 µl 2-propanol were used to separate the RNA from DNA and proteins. After washing with 75% ethanol, the dry RNA was dissolved in 50 µl of diethylpyrocarbonate (DEPC) water. To obtain cDNA, we reverse-transcribed 1 µg DNase-treated total RNA with oligo(dT) primers (0.01 µg/ml) in a reverse transcription-PCR mixture with a total volume of 20 µl. Quantitative PCR was performed using the Bio-Rad iCycler and SYBR Green. The following primers were used (5′-3′): GTACATCAAGACGGGAAGGC (forward) and AGTGTGCTCTGGGTGCTTCT (reverse) for IRAK-1, GTACATCAGACAGGGGAAACTTT (forward) and GACATGAATCCAGGCCTCTC (reverse) for IRAK-M, and ATGAGTATGCCTGCCGTGTG (forward) and CCAAATGCGGCATCTTCAAC (reverse) for β_2_ microglobulin (B2M) [9]. Quantification of the PCR signals for each sample was performed by comparing the cycle threshold values, in duplicate, for the gene of interest with the cycle threshold values for the B2M as housekeeping gene. All primers were validated according to the protocol. Mean relative mRNA expression was calculated using Pfaffl method. Values are expressed as a ratio of fold increase to mRNA levels of unprimed cells.

### Statistical analysis

The SPSS version 20.0 statistical software package was used to perform the calculations. In the cohort study, the differences between patient groups were analyzed using the chi-squared test or Fisher exact probability where appropriate, for categorical variables and the t-test for continuous variables. Adjustments for the effect of HbA1c and diabetic drug usage on outcome measures were performed using binary logistic regression. For the *in-vitro* experiments, results were pooled from at least 3 sets of experiments and analyzed, unless otherwise specified. The cytokine data was presented as mean±standard error of the means (SEM). The differences in cytokine production were tested using Mann-Whitney U test. The level of significance was set at p<0.05.

### Ethics statement

Ethics approval for the conduct of the above study had been attained through the Domain Specific Review Boards, National Healthcare Group, Singapore (no. 2012/00596 and no. 2012/00949). Adult study subjects had provided written informed consent to participate in the study. There were no study subjects under the age of 21 years old.

## Results

### Sulphonylurea usage was associated with increased in-patient complications during melioidosis

Of the 74 subjects with pre-existing diabetes on treatment and melioidosis, 44 (57.9%) were taking a sulphonylurea group drug either alone or in combination with other diabetic medications. The demographics and outcomes of this group of patients were studied against patients who did not receive sulphonylureas as part of their diabetic drug regimen. As described in Table 1, the background characteristics between the 2 groups of patients including immune status and renal function were similar. Glycaemic control as judged by their most recentHbA1c sugar control was similar between both groups (9.00 versus 9.59, p = 0.353).

**Table 1:** Baseline demographics of melioidosis patients on diabetic therapy.

	Sulphonylurea (N = 44)	Without Sulphonylurea (N = 30)	*P* value
**Gender**			
Male	39 (88.6%)	24 (80.0%)	0.336
Female	5 (11.4%)	6 (20.0%)	
Median age	58.2	56.5	0.536
Immunocompromised state	2 (4.5%)	4 (13.3%)	0.215
Statin use	22 (50.0%)	10 (33.3%)	0.155
HbA1c#	9.00±2.30	9.59±2.64	0.353
Serum creatinine (µmol/L)	182±200	201±281	0.750
Bp culture positive*	34 (77.3%)	26 (86.7%)	0.377
**Concurrent diabetic medication**			
Metformin	23 (52.3%)	14 (46.7%)	0.848
Insulin	3 (6.8%)	18 (60.0%)	0.001
Thiazolidinedione	1 (2.3%)	2 (6.7%)	0.562
α-glucosidase inhibitor	9 (20.5%)	0 (0%)	0.010
**Complications**			
Hypotension (inotropes)	13 (29.5%)	1 (3.3%)	0.005
Intubation	9 (20.5%)	2 (6.7%)	0.182
Renal Failure	8 (18.2%)	2 (6.7%)	0.187
Septicaemia	9 (20.5%)	3 (10.0%)	0.339
Any (above) complications	21 (47.7%)	5 (16.7%)	0.006
Length of stay (days)	27.7	21.5	0.168
Mortality	7 (15.9%)	1 (3.3%)	0.080

#10 (of 44) patients from the sulphonylurea group and 3 (of 30) patients from the no-sulphonylurea group had no recent HbA1c measurements available.

* *Burkholderia pseudomallei* culture positive. Other cases were diagnosed by positive melioidosis serology >1/64 titres.

However, patient receiving sulphonylurea-containing regimens had higher incidence of secondary end points indicating severity (47.7% versus 16.7%, p = 0.006). In particular, melioidosis patients on sulphonylurea were more likely to be hypotensive requiring inotropic support (29.5% versus 3.3%, p = 0.005). The other analysed criteria showed increased tendencies for mechanical ventilation and acute kidney injury requiring renal replacement therapy. Similarly there was a trend towards increased overall mortality in patients on sulphonylurea (15.9% versus 3.3%, p = 0.080). After adjustment for HbA1c, sulphonylurea usage remained a significant predictor of development of severity manifestations (adjusted odds ratio AOR 4.89, 95% confidence interval C.I. 1.37–17.5, p = 0.015). Mortality was not significantly linked to prior sulphonylurea exposure (AOR 4.48 95% C.I. 0.49–41.4, p = 0.186) even after HbA1c correction.

Of note, concurrent insulin therapy was prescribed more in diabetic regimens not containing sulphonylureas, and the converse for α-glucosidase inhibitor. Melioidosis patients who had insulin as part of their diabetic therapy had a lower incidence of severe manifestations during infection (19.0% versus 41.5%, p = 0.104). The use of metformin did not influence the clinical outcomes (data not shown).

### Sulphonylurea attenuates the inflammatory response

**Figure 1:**
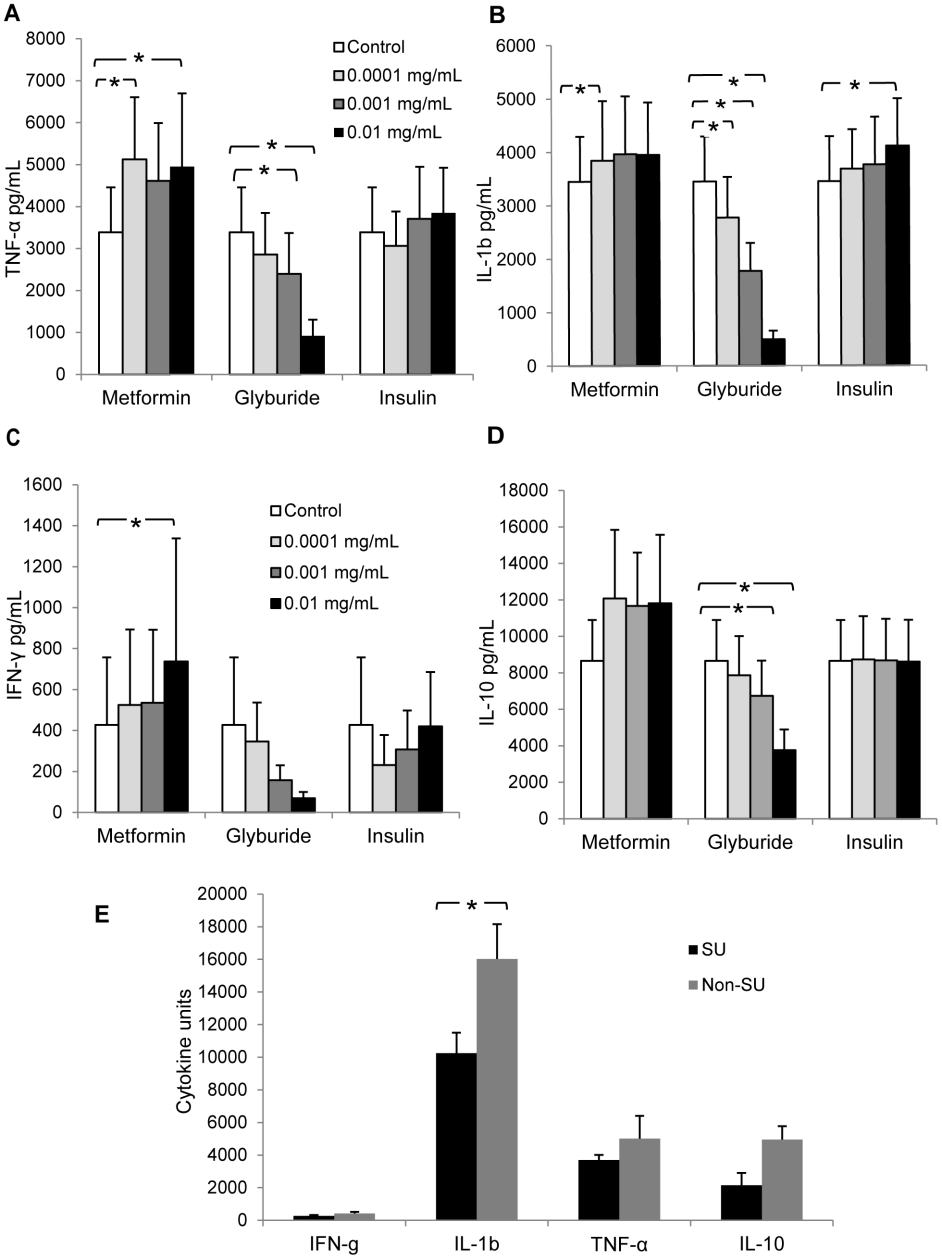
(A–D) Peripheral blood mononuclear cells (PBMC) from healthy donors were incubated with metformin, glyburide or insulin at the indicated doses (0.0001–0.01 mg/mL or control media) for an hour after which purified Bp antigen (1 µg/mL) was added for stimulation. (E) PBMC from diabetic patients receiving sulphonylurea (SU)-containing regimen (n = 9) versus those not on a non-SU regimen (n = 13) were stimulated with purified Bp antigen. Cytokine responses were measured at 24 h for TNF-α and IL-1b and at 48 h for IL-10 and IFN-γ. The *in-vitro* data are cumulative results of at least 3 sets of experiments (n≥9) and expressed as means±standard errors of the means (SEM). * p<0.05 compared to the respective control (white bar) for (A–D) or between SU and non-SU group for (E).

To specifically elucidate the influence of the respective diabetic drugs on the immune response, PBMC from healthy volunteers were primed with metformin, glyburide and insulin and then stimulated with purified Bp antigen. Over a drug concentration range of 0.001 mg/mL to 0.01 mg/mL, glyburide clearly diminished the production of TNF-α, IL-1β and IL-10 in a dose-dependent manner as compared to Bp control (in carrier medium) (Figure 1A–D). Conversely, insulin had a tendency to accentuate IL-1β response but this is only most evident at the dose of 0.01 mg/mL (Figure 1B). Low dose metformin had a limited effect in enhancing TNF-α, IL-1β and IFN-γ production (Figures 1A–C).

To further validate the above findings in the patient cohort, PBMC from diabetic patients were stimulated with purified Bp antigen. We found that patients who had sulphonylurea in their treatment regimen for diabetes had a significantly weaker IL-1β response (p = 0.04) as compared to patients who were on non-sulphonylurea containing regimens. The attenuative effects of sulphonylurea on the other cytokines in the patients were suggestive but not significant (Figure 1E).

### Sulphonylurea modulates cytokine mRNA transcription

**Figure 2:**
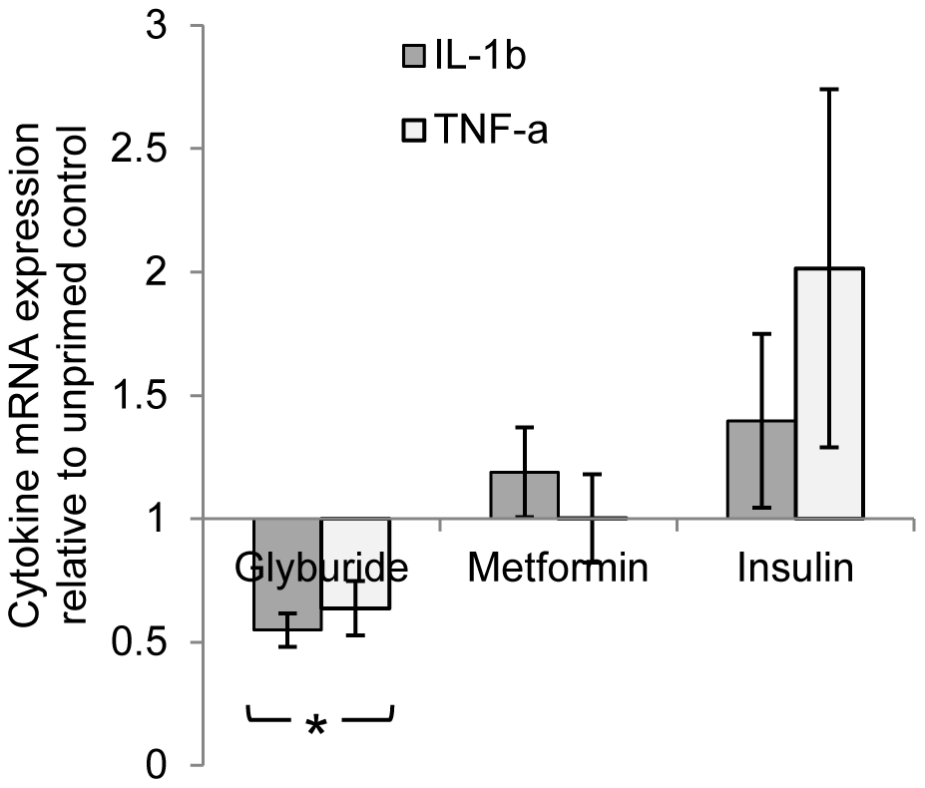
IL-1b and TNF-α mRNA transcriptions were suppressed in glyburide-primed PBMC as compared to metformin and insulin treatment. * p<0.05 relative to the ratio of fold increase in the mRNA levels of cells primed only in control media in absence of diabetic drugs.

Further to the above findings, we found that the attenuation of cytokine production by glyburide was effected upstream at the level of transcription. In the presence of glyburide, the IL-1β and TNF-α mRNA transcription was significantly reduced. In comparison, metformin and insulin induced minimal transcriptional changes (Figure 2).

### MyD88 and_JNK/ERK/p38 signaling are not influenced by sulphonylurea

**Figure 3:**
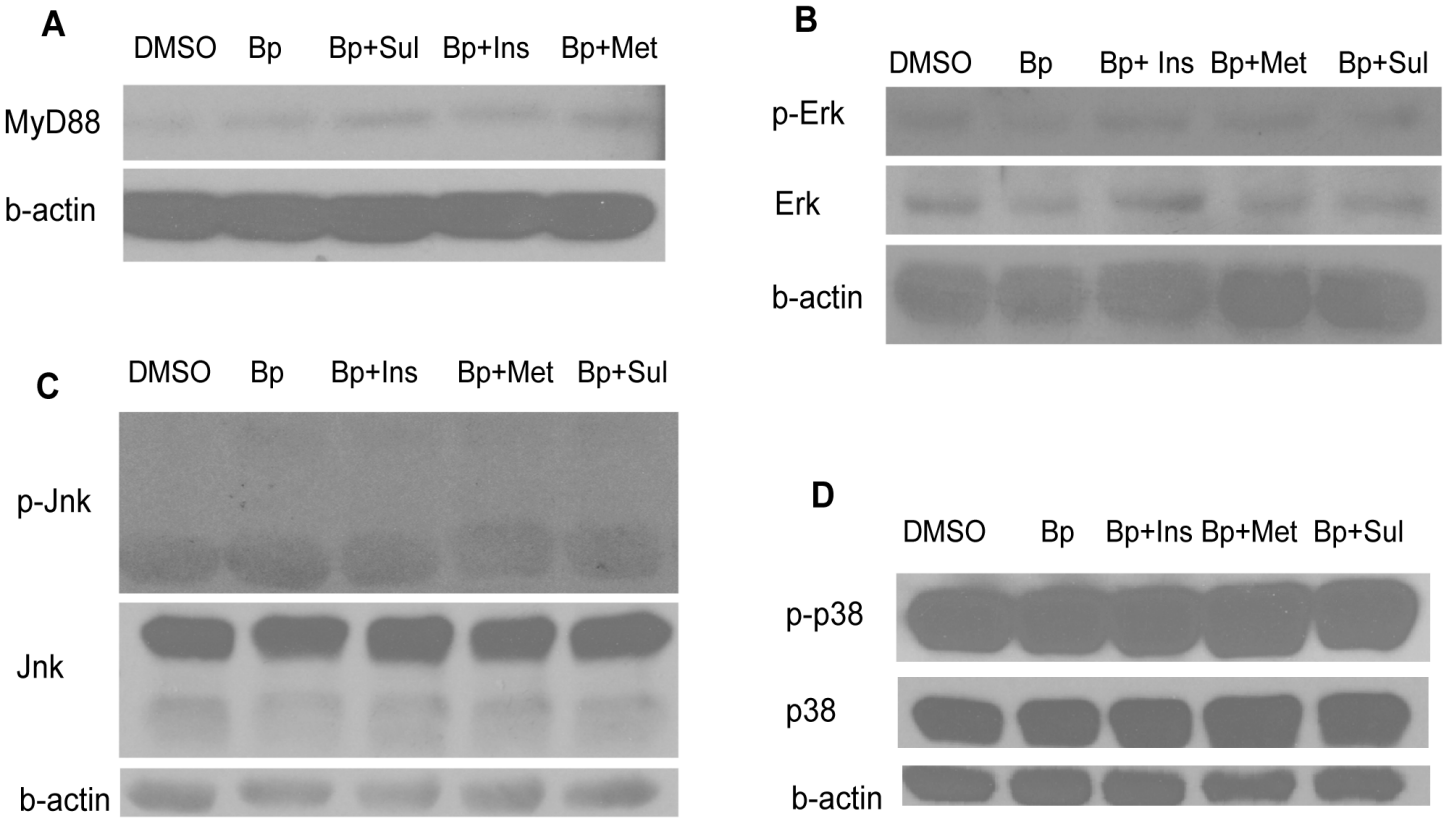
(A–D) Western blot from cell lysates of PBMC primed with diabetic drugs for 1 hour and then stimulated with purified Bp antigen for 30 minutes. The membranes were stripped and re-probed for MyD88, total and phosphorylated (p-) JNK, ERK and p38. Beta-actin was used as the loading control. Result is representative from 2 sets of experiment.

Because the suppressive effects induced by glyburide was generally seen with IL-1β, TNF-α and IL-10 and possibly not localized to a specific arm of the immune signaling pathway, we decided it was logical to study first, the major upstream adaptor molecule MyD88 and the mitogen-activated protein (MAP) kinases JNK, ERK and p38 in the canonical innate signaling pathways. However we found that the expression of MyD88, JNK, ERK and p38 were not affected glyburide-treated cells (Figures 3A–D).

### Sulphonylurea modulates immune response though IRAK-M

**Figure 4:**
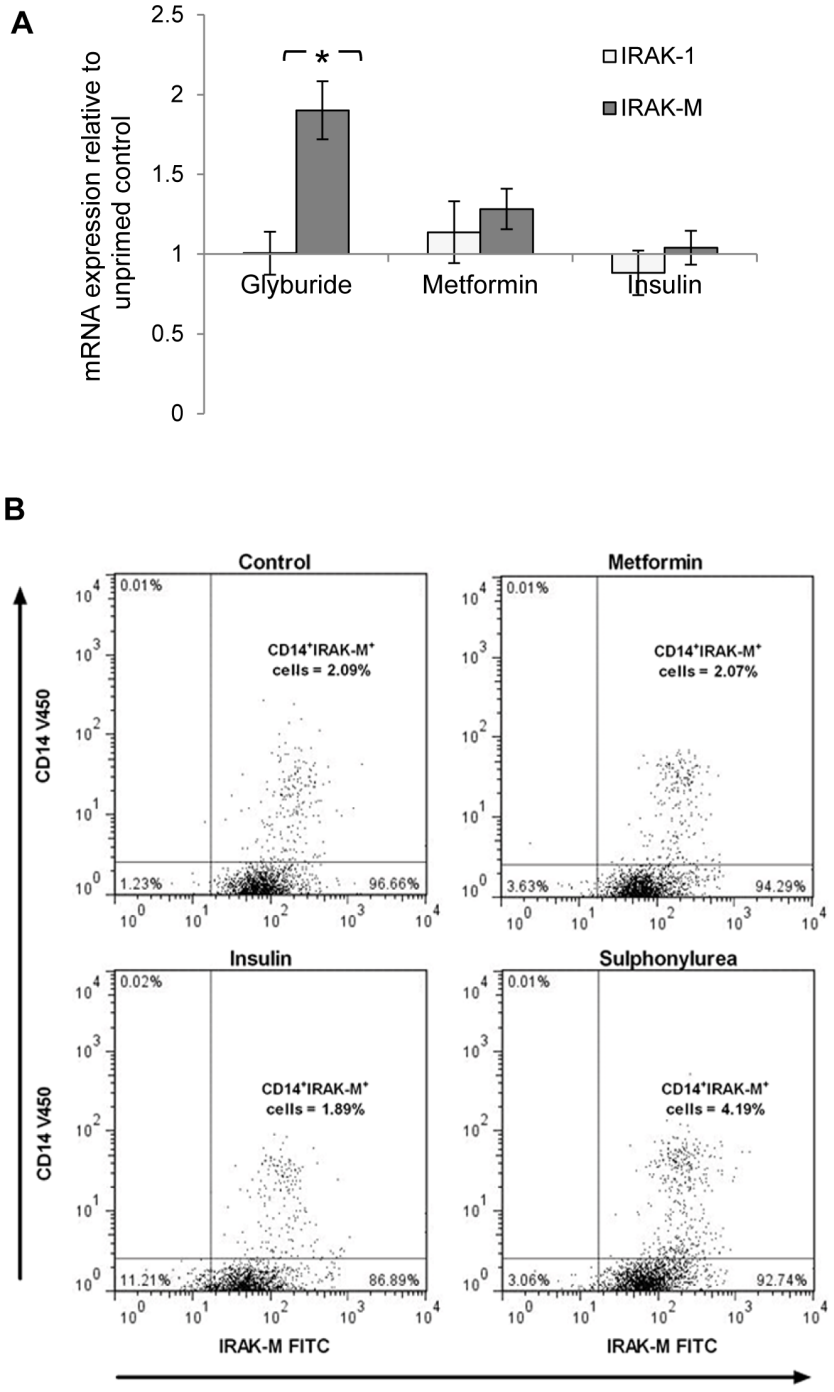
(A) IRAK-M mRNA expression is elevated by glyburide. * p<0.05 relative to the ratio of fold increase in the mRNA levels of cells primed only in control media in absence of diabetic drugs. (B) Flow cytometry of glyburide-treated PBMC showed increased expression of IRAK-M in CD-14 positive monocytes following stimulation with purified Bp antigen. The cells were surface labeled with anti-CD14-V450 antibody, permeabilized and then labeled with primary goat anti-IRAK-M antibody. Secondary labeling with donkey anti-goat-FITC antibody was performed and cells were analyzed by flow cytometry. Result from a representative set of experiment is shown.

Recently it has been proposed that elevated expression of IL-1R-associated kinase-M (IRAK-M), a regulator of the inflammatory signaling pathway, was associated with adverse outcome in septic melioidosis [9]. The role of IRAK-M in the context of diabetes treatment and melioidosis, however, is not known to date. We saw increased expression of IRAK-M mRNA in glyburide-treated cells. The interleukin-1 receptor-associated kinase 1 (IRAK-1) mRNA expression was not affected (Figure 4A). The effects of insulin and metformin on IRAK-M and IRAK-1 were limited. This was further confirmed on flow cytometery showing increased IRAK-M expression in monocytes exposed to glyburide (Figure 4B). This up-regulation of IRAK-M expression reasonably accounts for the immune suppressive effects induced by glyburide, and the association with disease severity observed with sulphonylurea use in melioidosis.

## Discussion

During an infection the capacity of the host to mount an appropriate immune and inflammatory response against the pathogen is pivotal. In the context of recent reports that diabetic medications like glyburide and metformin may possess inherent immune modulating capabilities, at least in the *in-vitro* setting [5], [6], we felt that it was important to study this further in the clinical context of melioidosis whereby diabetics are specifically predisposed to.

In this study, we found that the use of a sulphonylurea-containing diabetes treatment regimen is linked to a more severe clinical course especially hypotension during melioidosis. This finding has implications for the large number of diabetics living in melioidosis endemic regions, particularly Singapore, Thailand and Northern Australia. The low cost of sulphonylureas, its availability and its relatively wide therapeutic window contributes to the popularity of the drug. While glycaemic control between the 2 treatment groups might have been a potential factor contributing to the difference in outcomes, we have shown that HbA1c was not a significant confounder of the results.

We showed that glyburide had the capacity to suppress cytokine production. These effects were seen not only *in-vitro* in cells primed with the drug, but also similarly demonstrated in diabetic patients who were taking sulphonylureas. The importance of this latter finding is to be highlighted in that the differences in cytokine trends could still be elicited despite the numerous of confounders like multiple co-morbidities and polypharmacy in the clinical cohort. The attenuative effect on cytokine production was exerted at the level of mRNA transcription and one of the effects of glyburide was through the enhancement of IRAK-M which assumes a regulatory or inhibitory role in proinflammatory cytokine production in the TLR/IL-1R signaling pathway though inhibition of nuclear transcription factor NF-kappaB [14]. Conversely, IRAK-1 which up-regulates NF-kappaB, is suppressed. These findings on the mechanistic action of glyburide are novel as to date; glyburide is reported to act through the NALP3 inflammasome complex [5].

The response to infection by *B. pseudomallei* requires the host defence to first mount a prompt and effective innate immune response [7]. Results from our studies indicate that this capacity to mount an inflammatory cytokine milieu is compromised in the background presence of sulphonylureas in the body. This hypothesis is supported by the recent finding that increased IRAK-M with consequent immunosuppresion was associated with poor outcome in melioidosis, though the specific aspect of diabetes and drugs was not looked at in that study [9].

The findings of our study is in contrast to that by another group [10], who had reported reduced mortality, hypotension and respiratory failure with glyburide usage in melioidosis in Thailand. The group had hypothesized that the inhibition of the inflammasome and the neutrophil-mediated inflammatory process by glyburide might be beneficial in limiting sepsis-induced tissue injury and organ damage. Conversely, it is imperative that the host innate immunity be able to mount a robust inflammatory response against the pathogen during the initial phase of infection. The presence of sulphonylurea in the body compromises the immune response as we have demonstrated. Furthermore, such modulation of the IRAK complex as induced by sulphonylurea leads to susceptibility to infections as can be seen in patients with these genetic deficiencies [15]. In our smaller cohort of patients in Singapore, we were able to document diabetic drug history from our electronic records as well as the prescribed appropriate initial intensive therapy for at least 2 weeks followed by oral eradication therapy of at least 10 weeks as per recommended for melioidosis [16]. While HbA1c was not captured in the Thai cohort, we had sought to obtain the last known HbA1c of our patients and had factored glycaemic control into our analysis to show that sulphonylurea usage was an independent predictor of developing severe disease in melioidosis.

In our study, the diabetic patients who were receiving insulin tended not to be prescribed concurrent sulphonylurea. This was expected as per prescribing practice in the management of diabetes. Our data suggests that patients on insulin might have a lower risk of severe septic manifestations of melioidosis. However we are cautious in not over-interpreting these results at this point as functionally, the immunomodulatory effects of insulin are not as clearly evident as compared to the suppression of cytokines induced by sulphonylurea. To validate these findings, the mechanistic effects of insulin need to be elucidated further.

Diabetes mellitus remains the single most important risk factor for development of melioidosis. We find here that a sulphonylurea-containing diabetes treatment regimen suppresses the host inflammatory response and puts patients at higher risk for adverse outcomes. The implication of this finding may extend beyond melioidosis to other *Gram* negative septicaemia. Against the background of the popularity of sulphonylurea use in diabetes, this study highlights caution in the prescription of this class of drug especially in melioidosis-endemic regions.
